# TOPK promotes metastasis of esophageal squamous cell carcinoma by activating the Src/GSK3β/STAT3 signaling pathway via γ-catenin

**DOI:** 10.1186/s12885-019-6453-z

**Published:** 2019-12-30

**Authors:** Yanan Jiang, Jing Zhang, Jimin Zhao, Zhenzhen Li, Hanyong Chen, Yan Qiao, Xinhuan Chen, Kangdong Liu, Ziming Dong

**Affiliations:** 1 Department of Pathophysiology, School of Basic Medical Sciences, Zhengzhou University, Zhengzhou, 450001 China; 2 Henan Provincial Cooperative Innovation Center for Cancer Chemoprevention, Zhengzhou, 450001 China; 3 State Key Laboratory of Esophageal Cancer Prevention and Treatment, Zhengzhou University, Zhengzhou, 450052 China; 4 The Hormel Institute, Minnisota University, Austin, MN 55912 USA; 5 The China-US (Henan) Hormel Cancer Institute, Zhengzhou, 450008 China

**Keywords:** Esophageal squamous cell carcinoma, TOPK, Tumor metastasis, Src/GSK3β/STAT3, γ-Catenin

## Abstract

**Background:**

Esophageal squamous cell carcinoma (ESCC) is a fatal disease with poor prognosis. The predominant reason for ESCC-related death is distal metastasis. A comprehensive understanding of the molecular mechanism underlying metastasis is needed for improving patient prognosis. T-LAK cell-originated protein kinase (TOPK) is a MAPKK-like kinase, which plays a vital role in various physiological and pathophysiological processes. However, the role of TOPK in ESCC metastasis is unclear.

**Methods:**

Tissue array was used to evaluate the correlation between TOPK expression and ESCC lymph node metastasis. Wound healing assay, transwell assay, and lung metastasis mice model were used to examine the role of TOPK in the migration of ESCC cells in vitro and in vivo. Protein kinase array, mass spectrometry (MS), and molecular modeling were used to examine the pathways and direct target proteins of TOPK that are involved in ESCC metastasis. Additionally, immunofluorescence and western blotting analyses were performed to verify these findings.

**Results:**

The enhanced expression of TOPK was correlated with lymph node metastasis in the ESCC tissues. TOPK knockdown or treatment with the TOPK inhibitor (HI-TOPK-032) decreased the invasion and migration of ESCC cells in vitro. HI-TOPK-032 also inhibited the lung metastasis in ESCC cell xenograft in vivo model. Moreover, TOPK promoted the invasion of ESCC cells by activating the Src/GSK3β/STAT3 and ERK signaling pathways via γ-catenin.

**Conclusion:**

The findings of this study reveal that TOPK is involved in ESCC metastasis and promoted the ESCC cell mobility by activating the Src/GSK3β/STAT3 and ERK signaling pathways. This indicated that TOPK may be a potential molecular therapeutic target for ESCC metastasis.

## Background

Globally, esophageal cancer is the sixth leading cause of cancer-related death [1]. Esophageal cancer is classified as adenocarcinoma (EAC) or squamous cell carcinoma (ESCC) based on the histological examination [2]. In recent decades, there has been a drastic increase in EAC incidence in western countries. ESCC is the main histological type, which accounts for about 80% of esophageal cancer cases in China. Most patients with ESCC are diagnosed at advanced stages of the disease. The patients with ESCC exhibit a low 5-year survival rate (10–15%), which is mainly due to the lack of effective drugs [2]. Moreover, ESCC frequently metastasizes to the liver, lungs, regional lymph nodes, bone, and adrenal glands in the advanced stage [3].

Several wide-spread genomic alterations have been detected in ESCC using high-throughput sequencing technologies [2, 4]. There are 79 key genes that promote the development of ESCC. These genes are involved in 12 signal transduction pathways and are associated with genome stability, cell survival, and cell fate in ESCC. Recent studies revealed that the aberrant activation of various signaling pathways, such as PIK3CA and NOTCH1 pathways promote the development of ESCC and contribute to several processes of ESCC, such as metastasis and proliferation [2]. Therefore, there is an urgent need to identify an effective target in these pathways for the clinical treatment of ESCC. Additionally, the molecular mechanisms underlying ESCC metastasis must be elucidated. LAK cell-originated protein kinase (TOPK) is a serine-threonine kinase, which is involved in various physiological and pathological processes, such as cell cycle [5], apoptosis [6], cancer cell proliferation [7], and cell invasion [8]. Several studies have indicated that TOPK is an oncogenic kinase that is highly expressed in several types of human cancers, such as lung, colon, and ovary cancers. TOPK expression is positively correlated with the aggressive phenotype of these tumors [9, 10]. However, the expression and function of TOPK in ESCC metastasis are unclear. Hence, it is important to elucidate the underlying mechanism of TOPK in ESCC.

## Methods

### Chemical reagents

Tris, NaCl, and sodium dodecyl sulfate (SDS) used for molecular biology experiments and buffer preparation were purchased from Sigma-Aldrich. The following antibodies for western blotting analysis were purchased from Cell Signaling Technology: anti-Stat3 (#9139), anti-p-Stat3 (#9145), anti-Src (#2109), anti-p-Src (#12432), anti-Erk (#4696), anti-p-Erk (#4370), anti-GSK3β (#5676), and anti-p-GSK3β (#9327) antibodies. The anti-TOPK (ab236872), anti-p-TOPK (ab250790), and anti-γ-catenin (ab32572) antibodies were purchased from Abcam. The antibodies against β-actin (sc-47,778) and tubulin (sc-73,242) were purchased from Santa Cruz Biotechnology. The human phospho-kinase arrays were obtained from R&D Systems. RPMI-1640, Dulbecco’s modified Eagle’s medium (DMEM), and minimum essential medium (MEM) were purchased from BI (Bioind, Israel). Fetal bovine serum (FBS) was purchased from Gibico. The TOPK inhibitor, HI-TOPK-032 was a gift from Prof Zigang Dong. The stock solution of HI-TOPK-032 (50 mM) was prepared in dimethyl sulfoxide (DMSO) and stored at − 20 °C. The working solution of HI-TOPK-032 was prepared by diluting the stock solution to 1000 times and used for in vitro treatment.

### Human ESCC tissue array

The tissue microarray was purchased from Shanghai Outdo Biotech Co., Ltd. The information on clinical stages (stage I-IV) (AJCC 7.0) and TNM score was obtained from the patient clinical data. The tissue microarray comprises 10 paired ESCC and adjacent normal tissues with lymph node-negative tissue (N0), 19 N1 ESCC tissues, 10 N2 ESCC tissues, 8 N3 ESCC tissues with lymph node-positive tissue. The expression of TOPK in the ESCC metastasis tissue was evaluated by immunohistochemical staining. The tissue microarray was incubated with the anti-TOPK (1:50) antibody at 4 °C overnight. Next, the tissue microarray was washed and incubated with the secondary antibody. The proteins were detected using DAB. The positive cells were graded under a microscope (20 X) and analyzed using the Image J software.

### Cell culture

The following ESCC cell lines were purchased from Shanghai Xinyu Biological Technology Co., Ltd. (Shanghai, China): KYSE450 (XY-H600), KYSE510 (AD0604), KYSE140 (AD0602), KYSE70 (XY-H601), and KYSE30 (AD0601). These cells have been authenticated by STR technology of GENEWIZ Co., Ltd. (Suzhou, China) and no mycoplasma contamination. The KYSE450 cell line was cultured in DMEM supplemented with 10% FBS. The other ESCC cell lines were cultured in RPMI-1640 medium supplemented with 10% FBS, 100 μg/mL penicillin, and 100 U/mL streptomycin at 37 °C and 5% CO_2_.

### Western blot

The cells were lysed in the RIPA buffer [50 mM Tris-HCl (pH 7.4), 150 mM NaCl, 1 mM EDTA, 1% NP-40, 0.1% SDS, 0.5% sodium deoxycholate, 1 mM Na_3_VO_4_, and complete EDTA-free protease inhibitor (Roche)] for 20 min on ice. The cells were centrifuged and the supernatant was subjected to SDS-polyacrylamide gel electrophoresis (SDS-PAGE) using 10% gel. The resolved proteins were blotted onto a polyvinylidene difluoride membrane (Millipore, USA). The proteins were developed using the enhanced chemiluminescence (ECL) western blotting detection reagent (GE, USA) and analyzed using the LAS-1000UVmini image analyzer (GE, USA).

### Lentiviral infections

The lentiviral TOPK constructs, sh*TOPK*#1 (ATTAGTGCATACAGAGAAGAGTT) and sh*TOPK*#2 (GTCTGTGTCTTGCTATGGAAT) were gifts from Prof Zigang Dong. The lentiviruses were engineered by co-transfecting the shRNA-expressing vector with psPAX2 and pMD2.G constructs into the 293 T cells using jetPrime reagent. The viral supernatant was harvested and used to infect the cells along with 8 μg/mL polybrene. The cells were selected using 2 μg/mL puromycin to establish the stable KYSE510 and KYSE30 TOPK knockdown cell lines.

### Wound healing assay

For wound healing assays, the shMock-, sh*TOPK*#1-, and sh*TOPK*#2-transfected KYSE510 and KYSE30 cells were seeded on 6-well plates containing culture medium at 80% confluency and incubated for 24 h. The confluent monolayer was scratched using a fine pipette tip. The cells were washed twice with phosphate-buffered saline (PBS pH 8.0). The cell debris was removed and the cells were incubated with the growth medium containing 1% FBS. The migration of cells to the injured blank area was evaluated by capturing the images at 0, 24, and 48 h.

### Transwell migration assay

Transwell migration assay was performed using the transwell inserts for a 24-well plate. The upper chamber membrane (8 μm; Millipore) was coated with 100 μL of 200 μg/mL Matrigel (BD Biosciences, Franklin Lakes, NJ, USA) at 37 °C for 1 h before adding the cells. The shMock-, sh*TOPK*#1-, and sh*TOPK*#2-transfected KYSE510 and KYSE30 cells were plated in culture medium without serum or growth factors in the upper chamber of 1 × 10^5^ cells. The lower chamber contained medium supplemented with 20% FBS, which served as a chemo-attractant. After incubating for 24 h, the cells that did not migrate or invade through the pores were removed using a cotton swab. The cells on the lower surface of the membrane were fixed with methanol for 10 min and stained with 0.1% crystal violet. The cells were then counted in 5 randomly selected microscopic fields (× 100) in each well.

### Protein kinase array

The phosphorylated proteins were analyzed using the human phospho-kinase arrays, following the manufacturer’s instructions. Briefly, the cell lysates (500 μg) of shMock-, sh*TOPK#1*-, and sh*TOPK#2*-transfected KYSE510 cells were collected and incubated with each array at 4 °C overnight on a rocking platform shaker. The cell lysate was removed the next day and the arrays were washed thrice with washing buffer. The arrays were incubated with the secondary antibody for 2 h at room temperature (25 °C) and washed thrice with washing buffer. The array was incubated with horseradish peroxidase (HRP) solution on a rocking shaker for 30 min. The array was then washed thrice with washing buffer and the protein spots were visualized using a chemiluminescence detection kit. The density of each duplicated array spot was assessed using the Image J software (v.1.37v, NIH). The density of the sample was calculated after subtracting the background density and the density of negative control (PBS). The expression level in the sh*TOPK#1*- and sh*TOPK#2*-transfected cells was normalized (indicated as a value of 1) to that of the shMock-transfected cells.

### Surface plasmon resonance (SPR)

The glutathione-S-transferase (GST)-tagged TOPK proteins were purified from BL21 bacteria using the glutathione-sepharose 4B beads. The anti-GST antibody was immobilized onto the CM5 chip by amine coupling, following the manufacturer’s instructions (GE). The recombinant TOPK (Signalchem, T14-10G-10) and purified GST-tagged TOPK protein (> 90%) were captured by the anti-GST antibody on the CM5 chip. The KYSE510 cell lysate was introduced into the CM5 chip at a flow rate of 10 μL/min for 5 min with running buffer (1X kinase buffer, pH 7.2) for one cycle and the captured protein was recycled. The control group did not contain recombinant TOPK or GST-TOPK protein. The SPR procedure was performed at Bicore T200 (GE, USA).

### Nanoflow liquid chromatography-electrospray ionization-tandem mass spectrometry (LC-ESI-MS/MS)

The captured proteins bound to the recombinant TOPK, GST-TOPK (TOPK protein was purified by GST tag), and negative group (KYSE510 lysis buffer) in the SPR were subjected to mass spectrometry (MS). The samples were subjected to reduction by incubation with dithiothreitol (DTT) (final concentration 10 mmol/L) for 1 h at 37 °C. Next, the samples were subjected to alkylation by incubation with iodoacetamide (final concentration 30 mmol/L) at room temperature for 45 min. Finally, the samples were diluted with 25 mmol/L ammonium hydrogen carbonate buffer to ensure that the urea concentration was below 2 mol/L and incubated with sequencing grade trypsin at a mass ratio of 1:50 at 37 °C for 14 h. The LCS-ESI.MS/MS analysis was performed using freeze-dried samples dissolved in 60% acetonitrile (ACN) containing 0.1% formic acid. The sample was passed through a R3 self-loading desalting column conditioned with 99.9% acetonitrile (ACN) and 0.1% trifluoroacetic acid (TFA) twice. Next, the samples were subjected to gradient elution as follows: 0.1% TFA, 1% TFA, with 30% ACN, 60% ACN, 30% ACN. The MS/MS conditions were as follows: mobile phase, solution A (0.1% formic acid, 99.9% H_2_O), solution B (0.1% formic acid, 99.9% ACN); sample volume, 10 mL; continuous gradient elution; flow rate, 200 nL/min. The analytical column gradient elution settings for the LTQ Orbitrap elite and the MS parameters were as follows: setting mode, cation; collision model, collision-induced dissociation (CID); resolution, 120,000; mass range, 350–1800, before top 15 ionic strength. The data were analyzed using the Thermo Proteome Discoverer software (1.4.0.288) with the following settings: databases, Mascot and UniProt; enzyme, trypsin-P; maximum missed cleavage, 2; precursor mass tolerance, 10 ppm; fragment mass tolerance, 0.5 Da; fixed modifications, carbamidomethyl (c); variable modification, Gln > pyro-Glu (N-term q), acetyl (protein N-term), oxidation (M), Phospho (st), and Phospho (y); peptide confidence, high false discovery rate (FDR) < 0.01.

### Molecular modeling

To confirm whether TOPK binds to γ-catenin, in silico docking was performed using the Schrödinger Suite 2016 software. The TOPK and γ-catenin crystal structures were obtained from the Protein Data Bank [11] (PDB ID: 5J0A and 3IFQ, respectively) and prepared according to the standard procedures of the Protein Preparation Wizard (Schrödinger Suite 2012). The protein-protein docking of TOPK and γ-catenin was performed using the protein docking server with the interactive molecular graphics program [12]. The best configuration was selected to represent the binding mode.

### Immunofluorescence assay

Immunofluorescence assay was performed using the primary antibodies against TOPK and γ-catenin. The samples were then incubated with the fluorescein-conjugated affinity pure goat anti-mouse IgG or rhodamine (TRITC)-conjugated affinity pure goat anti-rabbit IgG secondary antibodies (1:2000) for 1 h. The samples were mounted on coverslips along with 4′, 6-diamidino-2-phenylindole (DAPI) (Santa Cruz TM). The images were captured and analyzed using high-throughput confocal microscopy (IN Cell Analyzer 6000, GE).

### Co-immunoprecipitation

For co-immunoprecipitation studies, the HEK293 cells were transfected with 4 μg pcDNA3-HA-TOPK and/or pDEST-myc-γ-catenin using jetPRIME reagent (Polyplus-transfection® SA, France) for 72 h. The protein was isolated in CHAPS buffer (as previously described) supplemented with protease inhibitor cocktail (Roche). The lysates were centrifuged at 14500 *g* for 30 min. Next, 500 μg protein in 500 μL was incubated with anti-hemagglutinin (HA) antibody overnight at 4 °C. The samples were then incubated with secondary antibodies (sc-2004, Santa Cruz) immobilized on A/G agarose (40 μL) for 4 h at 4 °C. The collected protein complexes were washed thrice with cold PBS and eluted by boiling in loading buffer at 95 °C, followed by incubation on ice for 2 min. The myc protein was resolved by SDS-PAGE and analyzed by western blotting.

### Lung metastasis in ESCC cell xenograft mouse models

The stable GFP-KYSE510 cells were established by transferring the pcDNA3.1-green fluorescent protein (GFP) vector and screened using G418. The GFP signal of KYSE510 cells was evaluated using the IVIS® Lumina III In Vivo Imaging System. Next, the GFP-KYSE510 cells (2 × 10^6^ cells/mL) were injected into the tail vein of BALB/c nude mice, which were purchased from Vital River, Beijing, China. After two weeks, these mice were divided into vehicle and treatment groups. The vehicle group was treated with 5% DMSO-PBS (*n* = 12, 6 females and 6 males), while the treatment group was injected with HI-TOPK-032 (*n* = 13, 7 females and 6 males). The mice in the treatment group were treated with HI-TOPK-032 was dissolved in 5% DMSO-PBS and was given to each mouse in treatment group once daily at 10 mg/kg (i.p., 100 μL per mouse). Four to five mice were kept in a pathogen-free environment with light controlled rooms (12 h cycles) and provided with food and water ad libitum. After two weeks, the mice were subjected to in vivo imaging. The fluorescence of GFP was evaluated as described previously [13]. The mice were euthanized by CO_2_ inhalation. The lung tumor tissues were used to evaluate the changes in the signaling pathways. This study was approved by the Ethics Committee of Basic Medical College of Zhengzhou University.

### Statistical analysis

All data are expressed as mean ± standard error (SEM) and all comparisons between samples were evaluated using the two-tailed non-parametric Mann-Whitney test and one-way or two-way analysis of variance (ANOVA) followed by Bonferroni post hoc test. The *p* values obtained from the tests are described in the Figure legends. Statistical significance is denoted as follows: * for *p* < 0.05, ** for *p* < 0.01, and *** for *p* < 0.001.

## Results

### TOPK was positively correlated with lymph node metastasis of ESCC patients

**Fig. 1:**
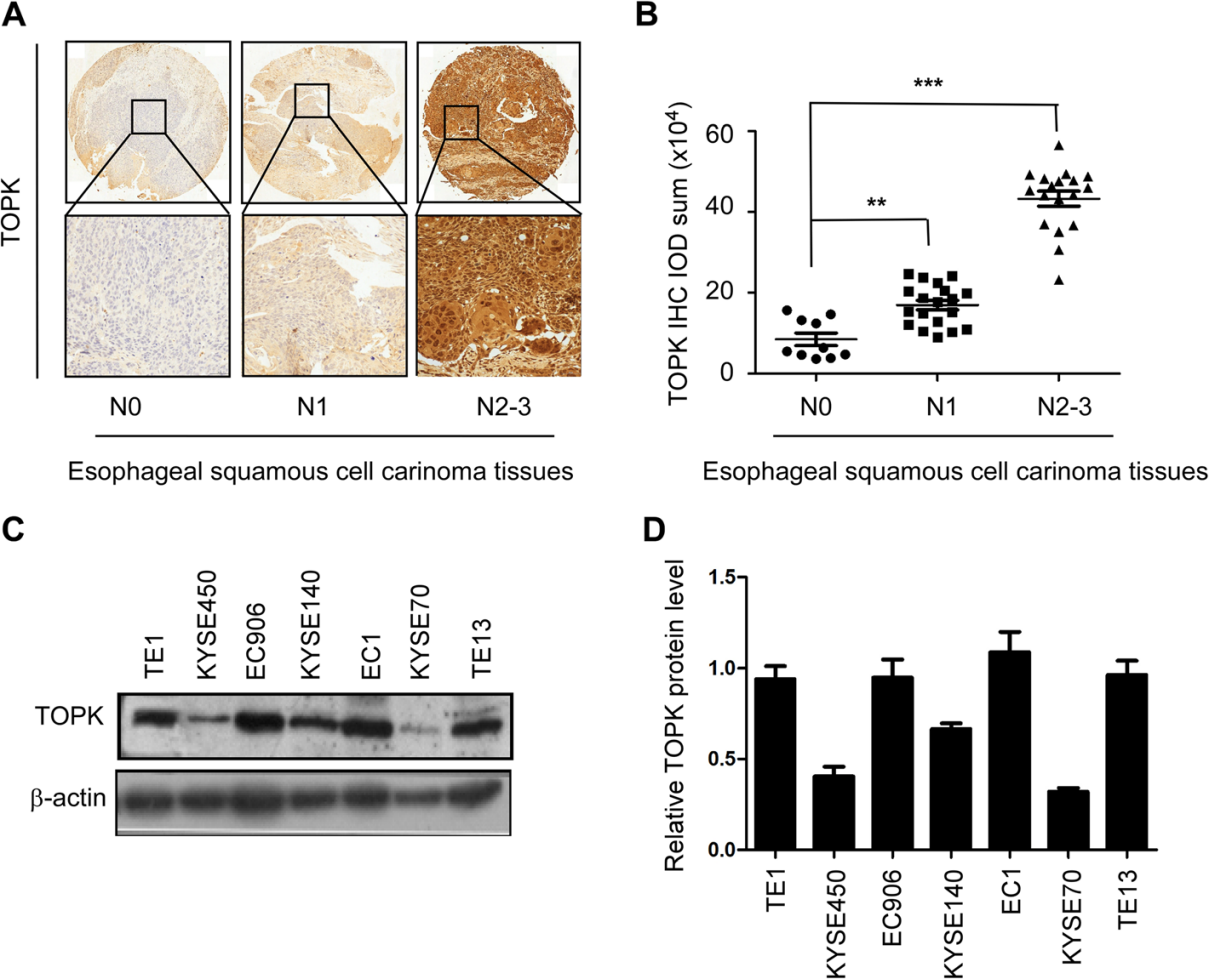
TOPK was positively correlated with lymph node metastasis in patients with esophageal squamous cell carcinoma (ESCC). **a**) The immunohistochemical (IHC) staining of TOPK in ESCC tissues exhibiting lymph node metastasis. TOPK expression in N0 (*n* = 10) (left panel), N1–2 (*n* = 19) (middle panel), and N3 ESCC tissues (*n* = 18) (right panel). Scale bar: 200 μm (upper) and 50 μm (down). **b**) The IHC staining analysis of TOPK expression in the N0, N1–2, and N3 ESCC tissues. **c**) Western blotting analysis of TOPK expression in different ESCC cell lines. d Relative expression of TOPK in different ESCC cell lines compared to the TE1 cell line. **p < 0.01, ***p < 0.001 vs N0 group

To investigate the clinical significance of TOPK in ESCC metastasis, the expression of TOPK was analyzed in the tissue samples of 49 patients with ESCC with or without the lymph node metastasis. The expression of TOPK was detected mainly in the cytoplasm and/or cell nucleus. The TOPK expression varied depending on the lymph node metastasis type (Fig. 1a). The TOPK expression level in the lymph node metastasis groups (N1 and N2–3 groups) was significantly (*p* < 0.001) higher than that in the no lymph node metastasis group (N0 group). This indicated a positive correlation between TOPK expression and lymph node metastasis (Fig. 1b). Additionally, the expression of TOPK varied in different ESCC cell lines. The expression levels of TOPK in the KYSE510, KYSE140, and KYSE30 cells were higher than those in the KYSE450 and KYSE70 cells (Fig. 1c-d).

### TOPK knockdown attenuated the migration and invasion of ESCC cells

**Fig. 2:**
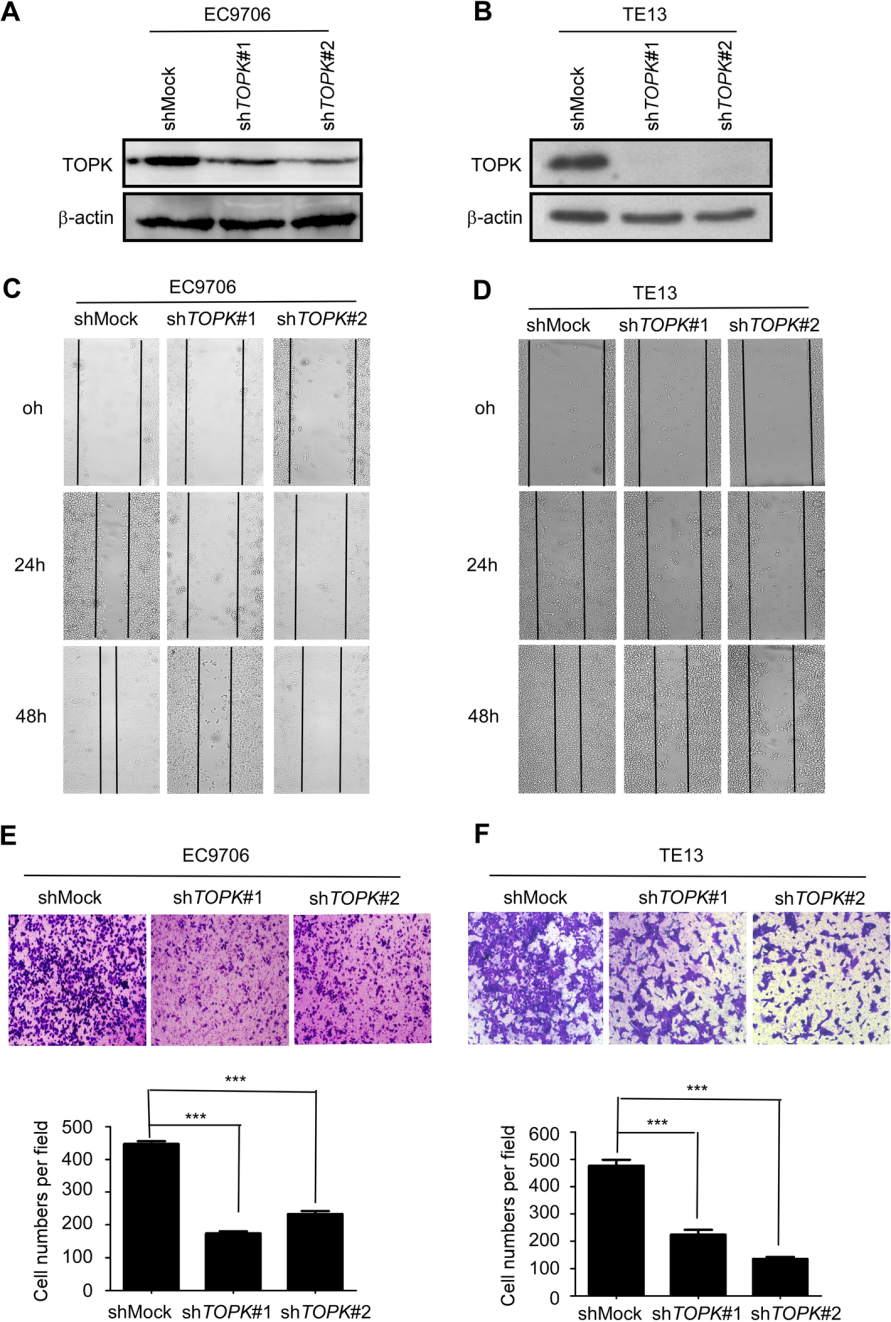
Knocking down TOPK attenuates the migration and invasion of esophageal squamous cell carcinoma (ESCC) cells. Knocking down TOPK expression in the KYSE510 (**a**) and KYSE30 cells (**b**) by lentiviral infection. Cell migration was evaluated at 0, 24, and 48 h in the stable TOPK knockdown cells (sh*TOPK*-transfected KYSE510 (**c**) and KYSE30 cells (**d**)) by wound healing migration assay. The invasion of KYSE510 (**e**) and KYSE30 (**f**) cells was measured by transwell migration assay after knocking down TOPK. ***p < 0.001 vs shMock group

**Fig. 3:**
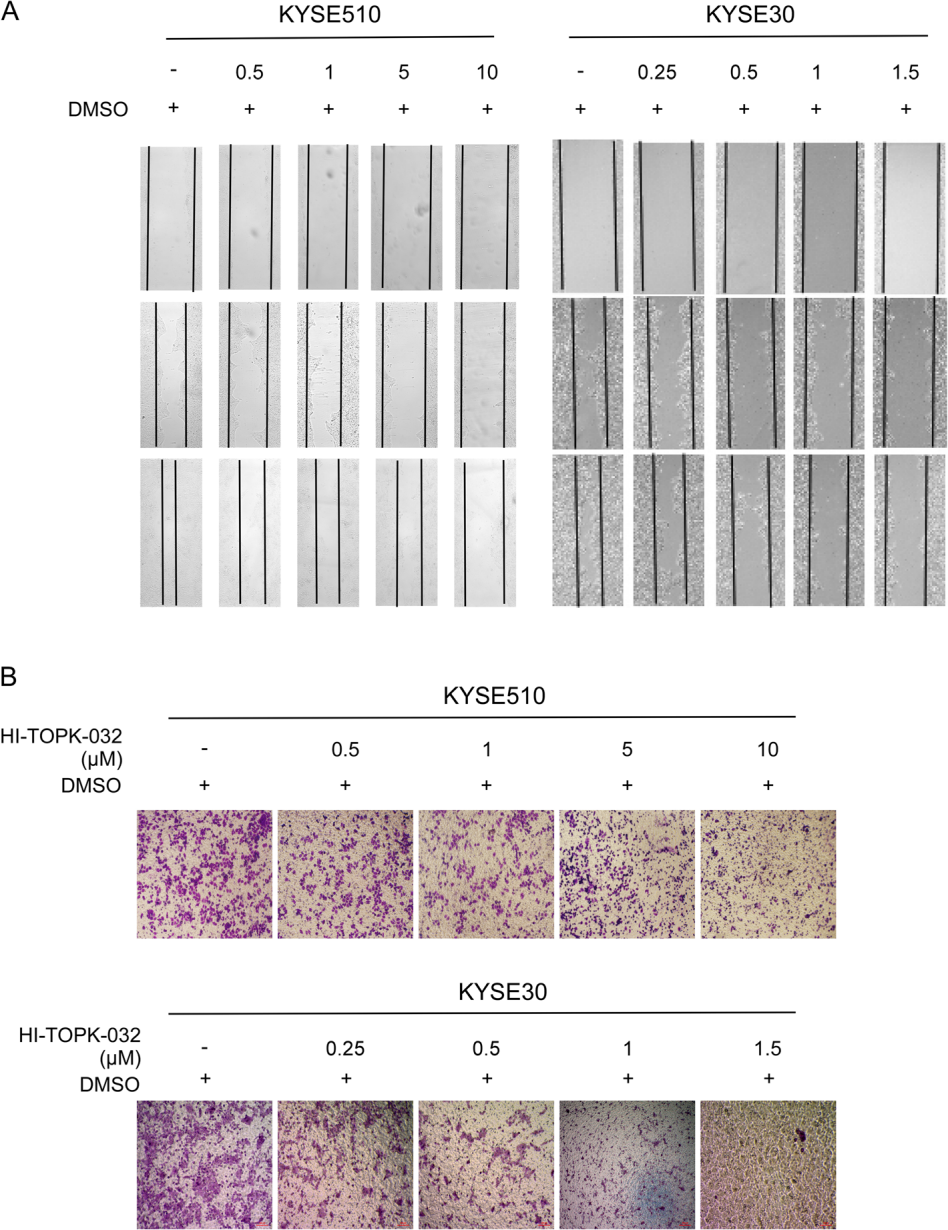
The TOPK inhibitor, HI-TOPK-032 attenuates migration and invasion of esophageal squamous cell carcinoma (ESCC) cells. The KYSE510 cell (**a**) was treated with 0,0.5,1,5 and 10 μM HI-TOPK-032, while KYSE30 cell (**b**) was treated at 0, 0.25, 0.5,1 and 1.5 μM HI-TOPK-032 for 0, 24, 48 h and the cell migration was analyzed by wound healing assay. The KYSE510 (**c**) and KYSE30 cells (**d**) were treated with different concentrations of HI-TOPK-032 and seeded in the upper transwell chamber. The number of migrated cell was counted in transwell migration assay

The TOPK knockdown stable cell lines were established by transfecting sh*TOPK*#1 and sh*TOPK*#2 into the KYSE510 (Fig. 2a) and KYSE30 (Fig. 2b) cells. The wound healing assay revealed that TOPK knockdown inhibited the migration of KYSE510 (Fig. 2c) and KYSE30 cells (Fig. 2d). The transwell migration assay indicated that TOPK knockdown decreased the invasion of KYSE510 (Fig. 2e) and KYSE30 (Fig. 2f) cells. The role of TOPK in the metastasis of ESCC was evaluated by treating the KYSE510 and KYSE30 cells with HI-TOPK-032, which inhibits the TOPK activity. Treatment with HI-TOPK-032 inhibited the migration of KYSE510 (Fig. 3a) and KYSE30 (Fig. 3b) cells. Moreover, HI-TOPK-032 dose-dependently decreased the invasion and migration of KYSE510 (Fig. 3c) and KYSE30 (Fig. 3d) cells after treatment for 24 h. These data suggested that TOPK was involved in ESCC cell mobility.

### Src/GSK3β/STAT3 and ERK signaling pathways were strongly inhibited by knocking down TOPK or by HI-TOPK-032 treatment

**Fig. 4:**
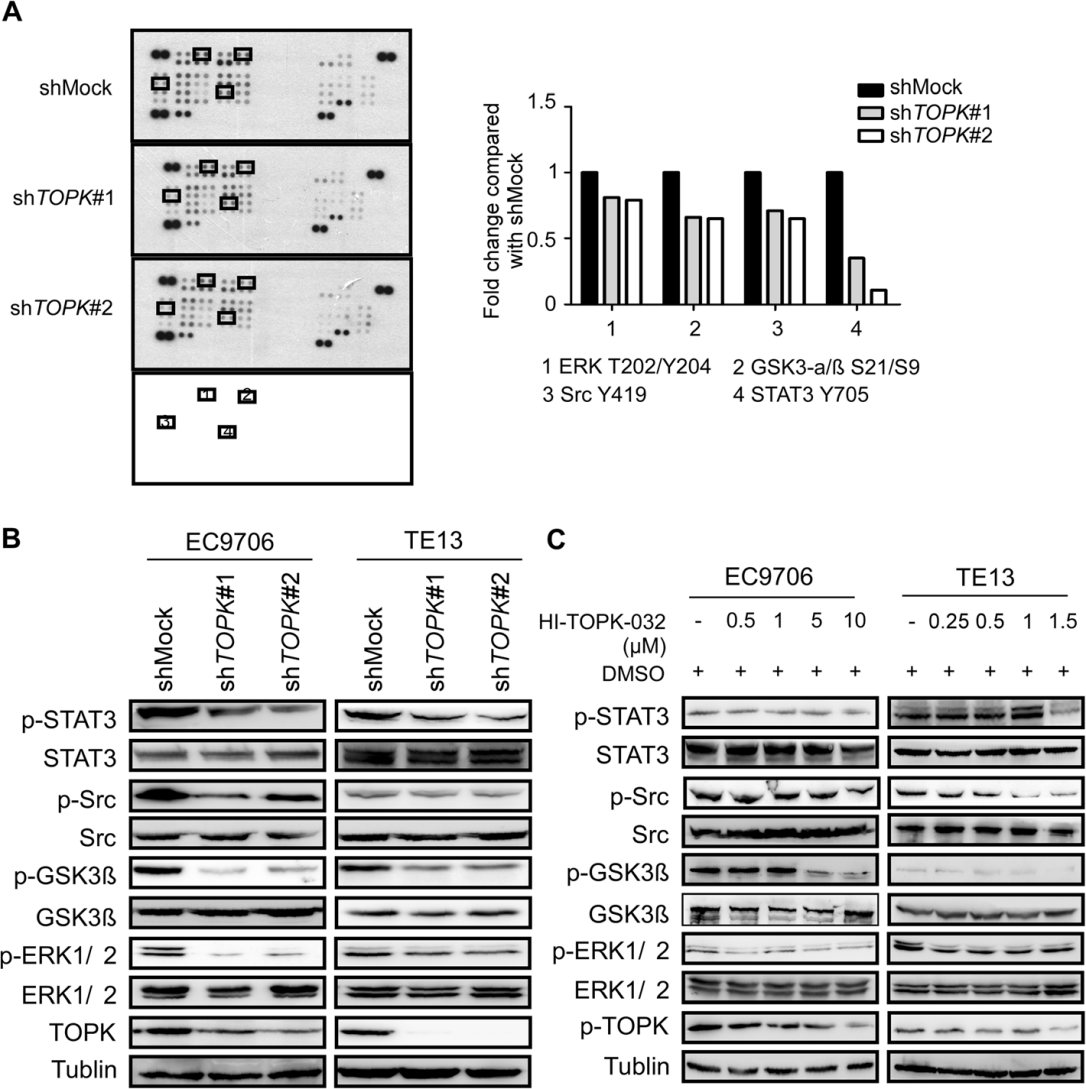
Src/GSK3β/STAT3 and ERK signaling pathways are strongly inhibited by knocking down TOPK and by treatment with HI-TOPK-032. **a**) The shMock-, sh*TOPK*#1- and sh*TOPK*#2-transfected cells were harvested and the TOPK level was determined by western blotting. Protein extracts (500 μg) were used for phospho-MAPK array analysis. The array spots were visualized using an enhanced chemiluminescence (ECL) kit (Fig a left). The density of each duplicated array spot (Fig a right) was measured as described in Materials and Methods. The graph showing the fold change in phosphorylation levels of Src, GSK3β, STAT3, and ERK normalized to the levels in the shMock-transfected cells (value of 1). The data are shown as the average of duplicate samples. **b**) The cell lysates (30 μg) from shMock0, sh*TOPK*#1-, and sh*TOPK*#2-transfected KYSE510 and KYSE30 cells were subjected to sodium dodecyl sulfate-polyacrylamide gel electrophoresis (SDS-PAGE) using 10% gel. The protein bands were visualized using a chemiluminescence detection kit after hybridization with horseradish peroxidase–conjugated secondary antibody. **c**) Western blot analysis of total and phosphorylated Src, GSK3β, STAT3, ERK and TOPK levels in the KYSE510 and KYSE30 cells, which were treated with different concentrations of HI-TOPK-032 for 24 h

The human phospho-kinase array (ARY003B, R&D) was used to examine the signaling pathway that mediates the TOPK-regulated ESCC metastasis. The effect of TOPK knockdown in the KYSE510 cells on the signal transduction pathways was evaluated using the human phospho-kinase array. Several signaling pathways were affected in the sh*TOPK#1*- and sh*TOPK#2*-transfected KYSE510 cells when compared with the shMock-transfected KYSE510 cells (Fig. 4a). The phosphorylation level of STAT3 Y705 markedly decreased to 0.35 and 0.11 in the sh*TOPK*#1- and sh*TOPK*#2-transfected KYSE510 cells, respectively. The phosphorylation levels of Src Y419, GSK3αβ kinase S21/S9, and ERK1/2 T202/Y204 in the sh*TOPK*#1-transfected KYSE510 cells decreased to 0.71, 0.65, and 0.81, respectively, whereas those in the sh*TOPK*#2-transfected cells decreased to 0.65, 0.66, and 0.79, respectively (Fig. 4a). These data indicated that TOPK might mediate the crosstalk between different signaling pathways and that TOPK played a complex role in ESCC metastasis. To further verify the phospho-kinase array results, the same cell lysates were subjected to western blotting analysis. The western blotting analysis revealed that the phosphorylation of STAT3 Y705, GSK3β S21, Src Y419, and ERK1/2 T202/Y204 was strongly inhibited upon TOPK knockdown in the KYSE510 and KYSE30 cells (Fig. 4b), which concurred with the array data. Next, the KYSE510 and KYSE30 cells were treated with different doses of HI-TOPK-032 for 24 h. The western blotting analysis revealed that HI-TOPK-032 dose-dependently inhibited the p-TOPK, STAT3, Src/GSK3β, and ERK signaling pathways (Fig. 4c). These findings indicated that TOPK may promote the invasion and migration of ESCC cells by activating several signaling pathways, such as the Src/GSK3β/STAT3 and ERK signaling pathways. This indicated that TOPK may mediate the crosstalk between various signaling pathways during ESCC metastasis.

### TOPK interacts with multiple proteins and binds with γ-catenin to enhance the invasion and migration of ESCC cell lines

**Fig. 5:**
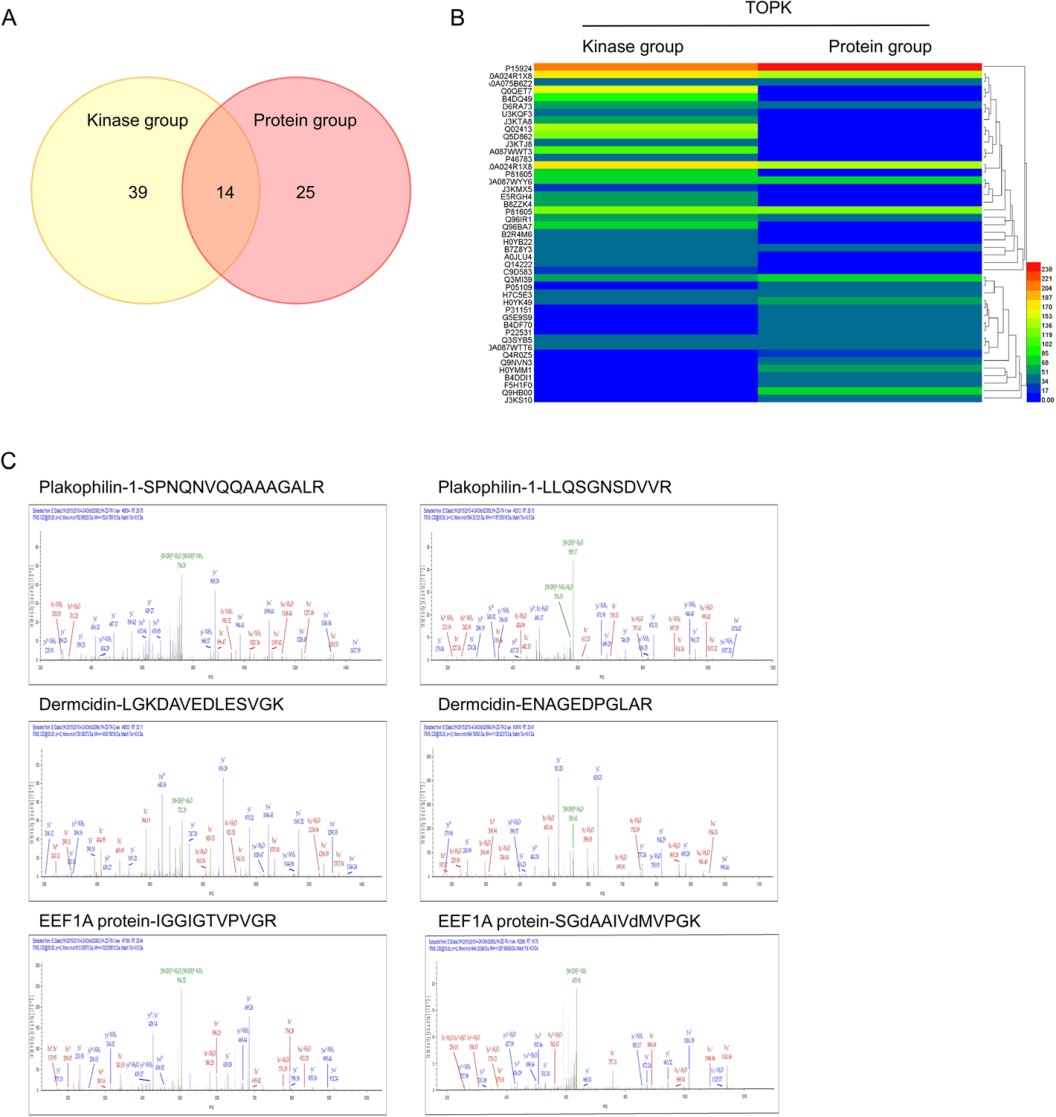
Mass spectrometry (MS) results of screening the proteins that bind with the glutathione-s-transferase (GST)-tagged TOPK protein and/or recombinant TOPK in the KYSE510 cell. **a**) Thirty-nine, twenty-five, and fourteen proteins bind to recombinant TOPK, GST-TOPK, and both, respectively. **b**) The quantitative analysis of proteins bound to recombinant TOPK and GST-TOPK. **c**) Representative proteins that bind with GST-TOPK protein and recombinant TOPK based on the Encyclopedia of Genes and Genomes: plakophilin-1, dermcidin, and EEF1A proteins

**Fig. 6:**
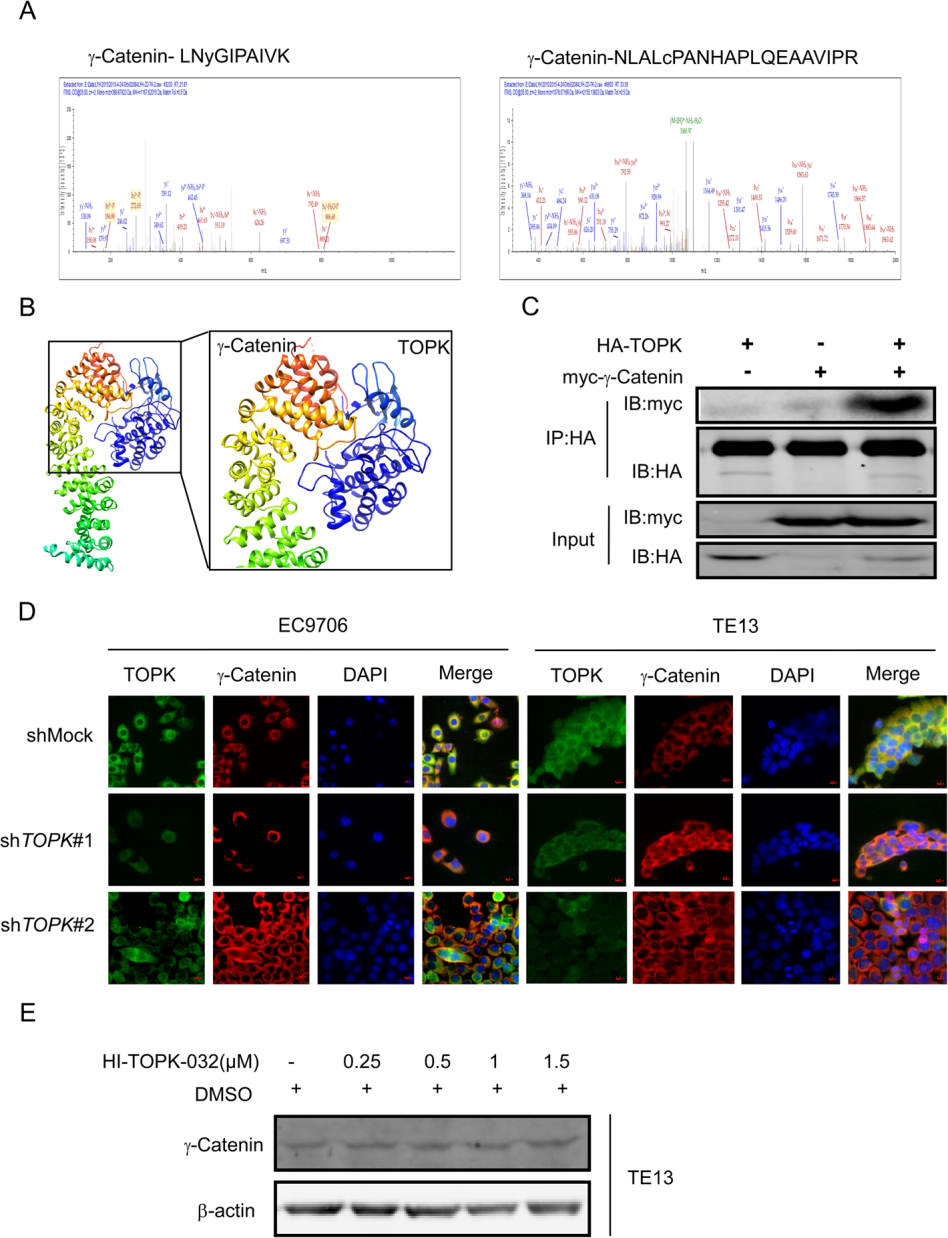
TOPK interacts with γ-catenin. **a**) The secondary mass spectrogram of γ-catenin, which binds with TOPK. **b**) Representative snapshot of TOPK-γ-catenin binding complex structure derived from MD simulations. TOPK and γ-catenin are represented in cyan and rainbow colors, respectively. **c**) TOPK binds with γ-catenin in vitro. pcDNA3- HA-TOPK and pDEST-myc-YBX1 were co-transfected into the HEK293 cells for 48 h, immunoprecipitated with anti-hemagglutinin (HA) antibody, and probed with secondary antibody immobilized on agarose beads, respectively. The expression of myc was analyzed by western blotting. **d**) Immunofluorescence analysis indicated that knocking down TOPK induced overexpression of γ-catenin in the KYSE510 and KYSE30 cells. **e**) Treatment with HI-TOPK-032 dose-dependently increased the expression of γ-catenin in the KYSE30 cells

SPR and MS were performed to screen the potential TOPK target proteins. The proteins with a score higher than 25 in MS analysis were chosen for further analysis. There were 53 and 39 proteins in the recombinant TOPK and GST-TOPK protein groups, respectively (Fig. 5a). Among these proteins, 39, 25, and 14 proteins bound to the recombinant TOPK, GST-TOPK, and both recombinant TOPK and GST-TOPK, respectively. Among the 14 proteins that bound to both recombinant TOPK and GST-TOPK, plakophilin-1, dermcidin, EEF1A had scores of 81.95, 65.91, and 41.6, respectively, and were associated with cell migration and invasion (Fig. 5b). Among the migration-related proteins, γ-catenin had the highest score (184.85) (Fig. 6a). Molecular modeling was also performed to understand the detailed binding interactions between TOPK and γ-catenin (Fig. 6b). The binding of TOPK to γ-catenin was evaluated by transfecting pcDNA3-HA-TOPK and/or pDEST-myc-γ-catenin into the HEK293 cell for 72 h. Immunoprecipitation analysis using the anti-HA antibody revealed that γ-catenin interacted with TOPK (Fig. 6c). Immunofluorescence analysis indicated that TOPK knockdown induced the expression of γ-catenin in the KYSE510 and KYSE30 cells (Fig. 6d). Furthermore, HI-TOPK-032 dose-dependently increased the expression of γ-catenin in the KYSE30 cells (Fig. 6e). These data demonstrated that TOPK interacts with several proteins, including γ-catenin, plakophilin-1, dermcidin, and EEF1A to promote the migration and invasion of ESCC cells.

**Fig. 7:**
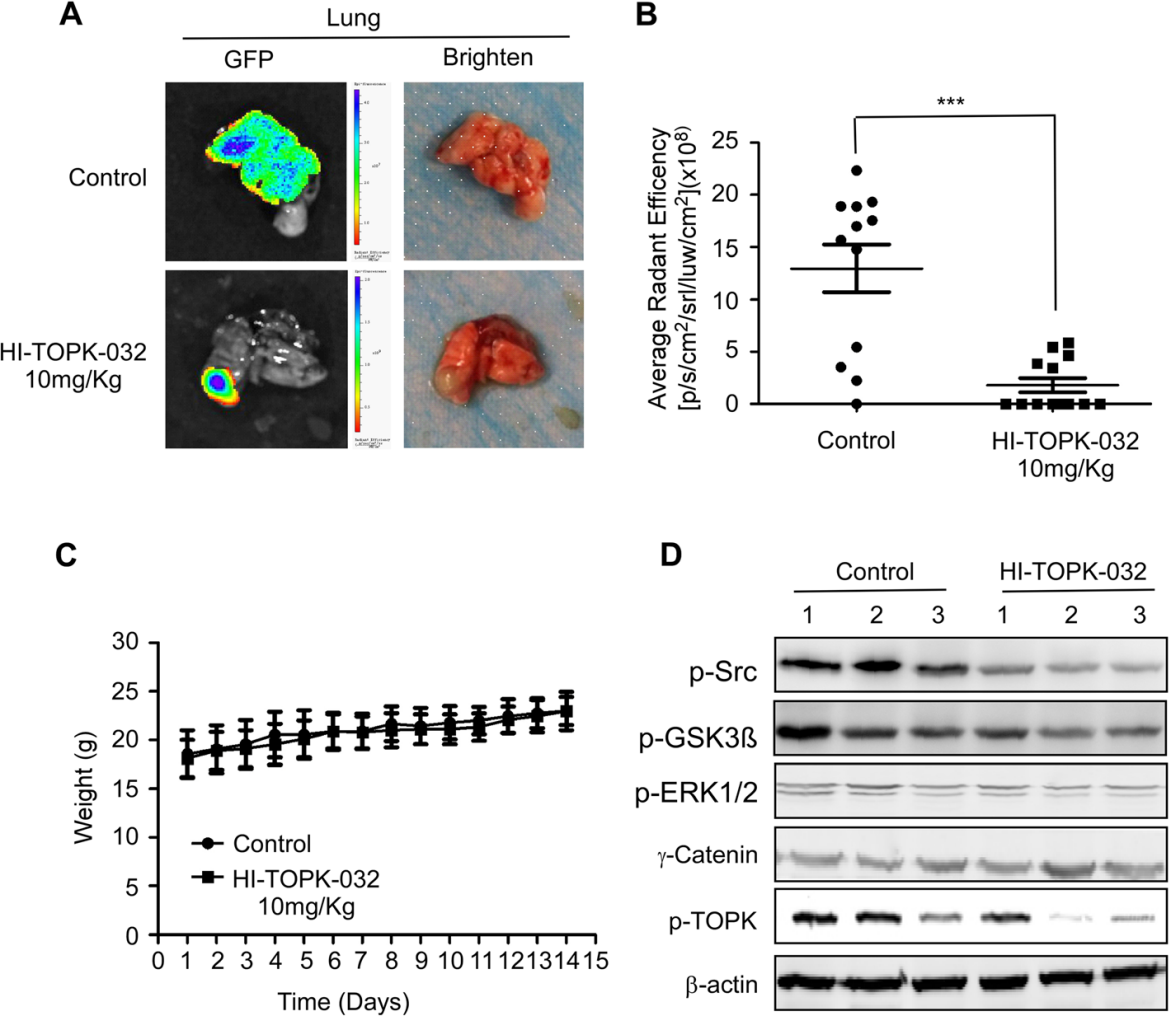
HI-TOPK-032 suppresses lung metastasis in the KYSE510 cell xenograft model. **a**) Representative images for lung metastasis in the vehicle and HI-TOPK-032 treatment group are shown. **b**) Compared to the vehicle-treated group, the HI-TOPK-032-treated group exhibited significant suppression of KYSE510 cell lung metastasis. The asterisks (***) indicate a significant (*p* < 0.001) decrease in green fluorescent protein (GFP) radiant efficiency compared to control. **c**) HI-TOPK-032 had no effect on the bodyweight of mice. **d**) Western blotting analysis was used to determine the levels of γ-catenin and the phosphorylation levels of TOPK, Src, GSK3β, and ERK in the lung HI-TOPK-032-treated and vehicle-treated groups

### The TOPK inhibitor, HI-TOPK-032 suppressed ESCC lung metastasis in a KYSE510 cell xenograft mouse model

Next, we evaluated the effect of TOPK on ESCC metastasis in vivo. Treating the athymic nude xenograft mouse model with HI-TOPK-032 suppressed lung metastasis without affecting the bodyweight in vivo (Fig. 7a-c). The western blotting analysis revealed that treatment with HI-TOPK-032 inhibited the expression of p-TOPK and the activation of Src, GSK3β, and ERK signaling pathways (Fig. 7d). This indicated that HI-TOPK-032 exerted an inhibitory effect on the metastasis of KYSE510 cells in the xenograft model by suppressing the Src, GSK3β, and ERK signal transduction pathways in vivo.

## Discussion

Metastasis is a complex multi-stage process and is the most lethal hallmark of ESCC [14]. Tumor metastasis involves enhanced cell motility. The cancer cells are transmitted from the primary tumor to the surrounding tissues and distant organs through the lymphatic and blood vessels. Clinically, tumor metastasis is mostly incurable and is the primary cause of cancer-related morbidity and mortality. Over 50% of ESCC-associated deaths are caused by distal metastasis, which is associated with poor prognosis, to the lymph nodes, lung, liver, bones, adrenal glands, and brain [15]. Therefore, identification of critical regulators that drive invasion and metastasis in ESCC will facilitate the development of new treatment strategies.

TOPK was first discovered in the lymphokine-activated killer T cells and is a branch of the MEK1/2 and MKK7 phylogenetic tree [16, 17]. Several studies have revealed a correlation between TOPK expression and poor prognosis in numerous cancers, such as leukemia [18], breast cancer [19, 20], ovarian cancer [21], cervical cancer [22], lung cancer [23], colon cancer [24], and oral cancer [9]. The enhanced expression of TOPK was reported to be associated with tumor aggressiveness, invasion, and metastasis [25]. Recent studies indicated that TOPK is a potential cancer-specific biomarker and a potential therapeutic target for cancer that can be used without markedly affecting the normal tissues [26]. However, there are no systemic studies that have evaluated the TOPK function in ESCC metastasis. In this study, the expression of TOPK in the N1–2 and N3 ESCC tissues was higher than that in the N0 ESCC tissues. The knockdown of TOPK inhibited the migration and invasion of ESCC cells. Additionally, treatment with HI-TOPK-032 inhibited the motility of ESCC cells in vitro and lung metastasis in vivo. These data indicated that TOPK overexpression was associated with ESCC metastasis and that the TOPK expression level may be a potential biomarker for ESCC. Therefore, it is important to explore the TOPK as a therapeutic target for ESCC.

Various studies indicated that the abnormal expression of TOPK in many cancers triggered aberrant activation of different signaling pathways, such as the ERK1/2, p38, c-Jun, and NF (nuclear factor)-κB signaling pathways [27]. In this study, the ERK signaling pathway was activated by TOPK, which concurred with the results of other studies. The results of this study also indicated that TOPK may mediate the crosstalk between several signaling pathways during ESCC metastasis, such as Src, GSK3β, and STAT3 signaling pathways. Shegan Gao et al. [28] reported that enhanced GSK3β expression was associated with higher metastasis rates and poor prognosis of ESCC. Additionally, the study demonstrated that STAT3 was a target of GSK3β using cancer phospho-antibody array and that enhanced GSK3β expression promoted ESCC progression through STAT3 in vitro and in vivo. These results indicated that GSK3β-STAT3 signaling could be a potential therapeutic target for ESCC treatment. However, Src was also overexpressed and functionally relevant to the progression of human ESCC. This indicated that Src might be a useful molecular target for ESCC prognosis and treatment [29]. Moreover, some studies demonstrated that Src-mediated Y216 GSK-3β phosphorylation and activation increased prostate cancer cell motility, proliferation, micrometastasis, and progression [30]. This suggested that GSK-3β activity can be modulated by Src kinases and that the pharmacological inhibition of Src-GSK-3β pathway may be a useful therapeutic strategy for prostate cancer. In this study, TOPK knockdown decreased the phosphorylation levels of Src, GSK3β, STAT3, and ERK proteins in the ESCC tissues. To the best of our knowledge, this is the first study to report the regulation of Src/GSK3β/STAT3 signaling pathway by TOPK, which is involved in the invasion process of ESCC in vitro and in vivo. Thus, TOPK may be vital for promoting the progression of ESCC. Therefore, TOPK as a therapeutic target may have unexpected effects by inhibiting other signaling pathways in ESCC.

To identify the direct target of TOPK that mediates the progression of ESCC, the proteins that bind to the purified TOPK protein and recombinant TOPK in the KYSE510 cell lysates were screened by SPR and MS. Several proteins bind to the recombinant TOPK, such as plakophilin-1, dermcidin, and EEF1A proteins. Among these TOPK-binding proteins, γ-catenin had the highest score. Therefore, we focused on evaluating the correlation between TOPK and γ-catenin. γ-catenin (also known as junction plakoglobin) is a desmosomal protein and may play a central role in desmosomes organization. Additionally, γ-catenin is required for the effective intermediate filament anchorage to desmosomes. γ-catenin was reported to be involved in cell invasion and metastasis of human oral squamous cell cancer, lung cancer, and bladder cancer [31]. Some studies have reported that γ-catenin functions as a tumor suppressor in ESCC and that the γ-catenin expression levels affected the overall survival. This indicated that low γ-catenin was associated with lymph node metastasis and may serve as a prognostic marker in human ESCC [32]. The results of this study demonstrated that TOPK knockdown or treatment with HI-TOPK-032 rescued the expression of γ-catenin in the ESCC cells. Moreover, TOPK directly binds to γ-catenin, which results in the dissociation of desmoplakin. Therefore, this suggested that downregulating TOPK may increase and stabilize γ-catenin and subsequently decrease the cell adhesion and motility.

Similar to its homolog β-catenin, γ-catenin is also reported to be involved in several cell signaling cascades during cell motility, cell proliferation, and apoptosis [33]. All these processes involve the interaction of cytoplasmic γ-catenin with various kinases and the inhibition of Wnt − β-catenin signaling by nuclear γ-catenin. The silencing of γ-catenin but not desmoplakin is reported to activate the p38-dependent keratin filament collapse and cell dissociation [34]. However, the signaling mechanisms underlying the tumor suppressor activity of γ-catenin in ESCC cells are not well understood. Recent studies demonstrated that γ-catenin knockout cells exhibited enhanced activation of Src signal pathways and that γ-catenin regulated cell motility through distinct Src and Rho GTPase-dependent pathways [35]. Therefore, decreased γ-catenin may be the primary reason for activating the Src signal pathways during ESCC metastasis.

**Fig. 8:**
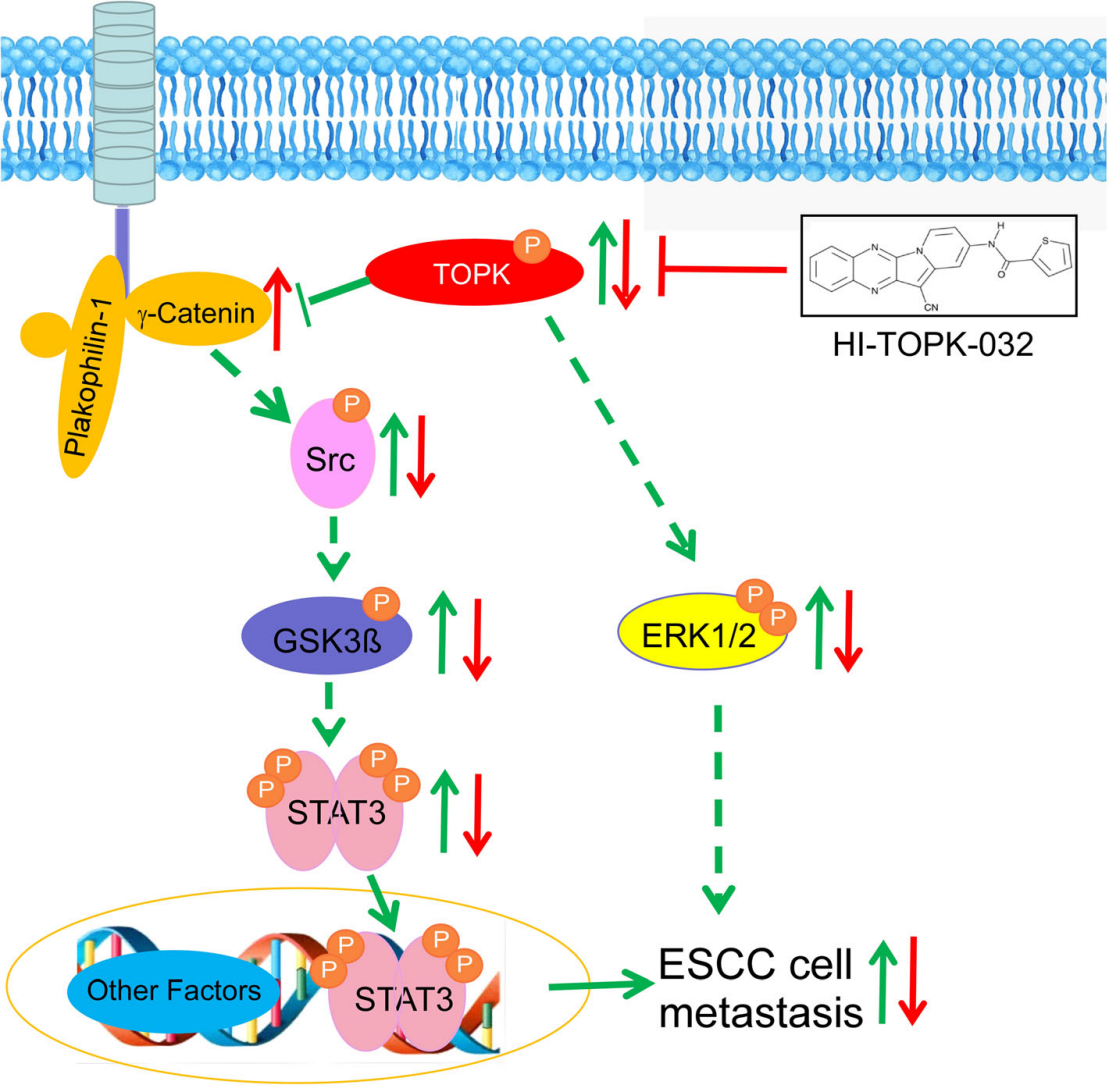
Schematic model depicting the promotion of esophageal squamous cell carcinoma (ESCC) cell metastasis. TOPK binds to γ-catenin and the deregulated γ-catenin activates the Src/GSK3β/STAT3 signaling pathway. TOPK also activates the ERK and Src/GSK3β/STAT3 signal pathway. These two activated pathways increase the cancer cell invasion and mobility and subsequently promote ESCC metastasis. Further, the TOPK inhibitor, HI-TOPK-032 inhibits the activated Src/GSK3β/STAT3 and ERK signaling pathways resulting in decreased ESCC metastasis

The following model illustrates the promotion of ESCC cell metastasis by TOPK (Fig. 8): TOPK binds γ-catenin and the deregulated γ-catenin activates the Src/GSK3β/STAT3 signaling pathway. This enhances the ESCC metastasis by promoting cell invasion and mobility. TOPK may also activate the ERK signal pathway to promote metastasis. Interestingly, the TOPK inhibitor, HI-TOPK-032 also inhibits the ESCC cell metastasis through the same Src/GSK3β/STAT3 signaling pathway. However, the molecular mechanisms underlying the promotion of ESCC cell metastasis by high levels of TOPK must be investigated in the future.

## Conclusion

TOPK is involved in ESCC metastasis and is a potential molecular therapeutic target for ESCC. This study demonstrated that TOPK promotes the metastasis of ESCC cells by activating the Src/GSK3β/STAT3 signal pathway via γ-catenin.

## Data Availability

All data generated or analyzed during this study are included in this published article.

## Abbreviations

ESCC: Esophageal squamous cell carcinoma
IHC: immunohistochemical staining
MS: mass spectrometry
SEM: standard error of mean
SPR: surface plasmon resonance
STAT3: signal transducer and activator of transcription 3
TOPK: T-LAK cell-originated protein kinase
