# Correction to: TOPK promotes metastasis of esophageal squamous cell carcinoma by activating the Src/GSK3β/STAT3 signaling pathway via γ-catenin

**DOI:** 10.1186/s12885-020-6514-3

**Published:** 2020-01-20

**Authors:** Yanan Jiang, Jing Zhang, Jimin Zhao, Zhenzhen Li, Hanyong Chen, Yan Qiao, Xinhuan Chen, Kangdong Liu, Ziming Dong

**Affiliations:** 1 Department of Pathophysiology, School of Basic Medical Sciences, Zhengzhou University, Zhengzhou, 450001 China; 2 Henan Provincial Cooperative Innovation Center for Cancer Chemoprevention, Zhengzhou, 450001 China; 3 State Key Laboratory of Esophageal Cancer Prevention and Treatment, Zhengzhou University, Zhengzhou, 450052 China; 4 The Hormel Institute, Minnisota University, Austin, MN 55912 USA; 5 The China-US (Henan) Hormel Cancer Institute, Zhengzhou, 450008 China

**Correction to: BMC Cancer**

**https://doi.org/10.1186/s12885-019-6453-z**

Following publication of the original article [1], the authors reported errors in Fig. 1 C and D, Fig. 2, Fig. 4 B and C and Fig. 6 D and E.

In Fig. 1 C and D, the cell lines are different, it should be as follow:

c) Western blotting analysis of TOPK expression in different ESCC cell lines. d) Relative expression of TOPK in different ESCC cell lines compared to the KYSE450 cell line.

The updated Fig. 1 C and D is supplied below. 

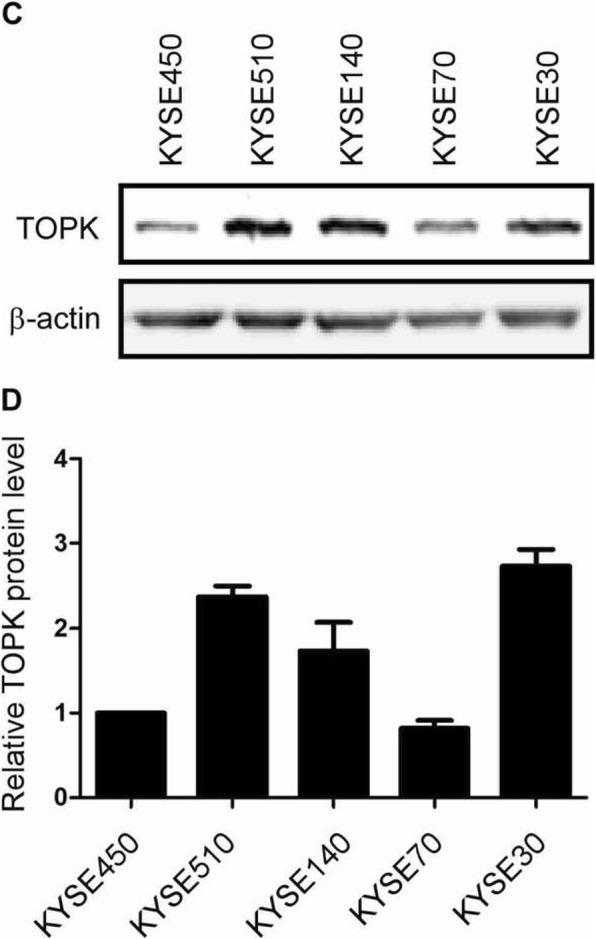



In Fig. 2, Fig. 4 B and C, Fig. 6 D and E, the cell lines were mislabeled. EC9706 should be instead of KYSE510, TE13 should be instead of KYSE30.
